# Post-Ingestive Sensations Driving Post-Ingestive Food Pleasure: A Cross-Cultural Consumer Study Comparing Denmark and China

**DOI:** 10.3390/foods9050617

**Published:** 2020-05-11

**Authors:** Mette Duerlund, Barbara Vad Andersen, Kui Wang, Raymond C. K. Chan, Derek Victor Byrne

**Affiliations:** 1 Food Quality Perception and Society, Department of Food Science, Aarhus University, Agro Food Park 48, 8200 Aarhus N, Denmark; barbarav.andersen@food.au.dk (B.V.A.); derekv.byrne@food.au.dk (D.V.B.); 2 Neuropsychology and Applied Cognitive Neuroscience Laboratory, CAS Key Laboratory of Mental Health, Institute of Psychology, Chinese Academy of Sciences, Beijing 100049, China; wangk@psych.ac.cn (K.W.); rckchan@psych.ac.cn (R.C.K.C.); 3 Department of Psychology, University of Chinese Academy of Sciences, Beijing 100049, China

**Keywords:** cross-cultural, post-ingestive food pleasure, food reward, post-ingestive sensation, satisfaction, china, Denmark

## Abstract

Culture is one of the main factors that influence food assessment. This cross-cultural research aimed to compare Chinese and Danish consumers in their post-ingestive drivers of Post-Ingestive Food Pleasure (PIFP). We define PIFP as a “subjective conscious sensation of pleasure and joy experienced after eating”. We conducted two in-country consumer studies in Denmark (*n* = 48) and in China (*n* = 53), measuring post-ingestive sensations and PIFP using visual analogue scale, for three hours following consumption of a breakfast meal. Key results revealed perceived Satisfaction, Mental, Overall and Physical wellbeing to be highly influential on PIFP in both countries. Moreover, Danish consumers perceived appetite-related sensations such as Satiety, Hunger, Desire-to-eat and In-need-of-food to be influential on PIFP, which was not the case in China. In China, more vitality-related sensations such as Energized, Relaxation and Concentration were found to be drivers of PIFP. These results suggest similarities but also distinct subtleties in the cultural constructs of PIFP in Denmark and in China. Focusing on Food Pleasure as a post-ingestive measure provides valuable output, deeper insights into what drives Food Pleasure, and, importantly, takes us beyond the processes only active during the actual eating event.

## 1. Introduction

Culture is one of the main underlying factors that influence how we assess food, our attitudes and beliefs about food and our food choices [1]. Considering the globalization and emergent food markets, where foods are exported beyond national borders, we need to take into consideration cultural aspects when seeking to understand human eating behaviors on the respective markets [2]. Consequently, cross-cultural research has become increasingly more pertinent within Sensory and Consumer Science [3,4], and several studies suggest that cultural differences exist in the way we perceive food, in our associations with specific foods, and also within food-related concepts [5,6,7,8,9,10,11]. The contribution and importance of cross-cultural research studies thus bring new perspectives in the domain of Sensory and Consumer Science, and it contributes to the development and understanding of various food concepts. 

Emphasis on cultural differences in food perceptions might be imperative in our association with Food Reward. Food Reward comes in many disguises and the concept can be defined and explained in many different ways. Reward is not a unitary construct, but comprises multiple psychological components [12], and different disciplines include different measurements of Food Reward. Investigating reward initially originates from disciplines such as psychology and neuroscience, where activation of brain circuits and neural pathways bring important knowledge. Reward research has been used to provide insights into several psychological and cognitive conditions including drug addictions, depression, eating disorders, gambling, obsession, sex addiction etc. [12,13]. Neuroscientists disclose three major components of reward namely Motivation, Learning and Affect. Each category comprises both explicit and implicit psychological constituents [12,14]. Motivation includes wanting, either as a conscious desire for incentives or as an underlying implicit motivation for reward. Learning represents association and prediction of future rewards based on experience, which includes both explicit cognitive expectancy and implicit associative conditioning. Affect includes liking and pleasantness, either as an implicit affect response or as a conscious pleasure in the ordinary sense of the term [13]. The present research situates and considers Post-Ingestive Food Pleasure (PIFP) as part of the Affect category with focus on conscious pleasure, inspired and described by [12,14].

Food Reward functions as an important conception for research within disciplines such as Health, Nutrition and Food Sciences [15]. For instance, several researchers within the Sensory and Consumer Science field seek to better understand Food Reward’s role in appetite and hence our eating behaviors [16]. The most common Food Reward measures are self-reported liking [16,17], self-reported desire-to-eat [13] or wanting for a specific food [13,16]. Many Food Reward measures and tasks have been developed and are in active use [18]. Rogers and Hardman (2015) define Food Reward as the “momentary value of a food to the individual at the time of ingestion”, measured directly with a rating of “desire to eat the entire portion right now” [19]. This approach and definition have also been used by, e.g., Ruddock et al. (2017) [19,20]. Explicit liking and desire to eat are most frequently evaluated using rating scales such as visual analogue scales [16]. The Leeds Food Preference Questionnaire (LFPQ) is a computer based measurement tool for Food Reward first proposed by Finlayson et al. (2007) [21]. LFPQ utilizes food image stimuli to evaluate explicit liking and wanting as well as implicit wanting in a choice task. The LFPQ has been applied and/or adapted by several researchers [16,20,22,23,24]. Additional Food Reward measures include grip force operant tasks [18,20,25], willingness to pay [20,26], and Emotional attentional blink [18,27]. An operant task could include tapping a space bar for 60 seconds, being told, the more you tap, the more of a given food you will receive [19,20], or by squeezing a handheld dynamometer as a response to specific food images [25]. 

The majority of the studies investigating Food Reward within the appetite space, measure and apply liking, wanting and other reward-associated tasks prior to or during consumption, so as part of the momentary eating event [17]. However, Food Reward derived from eating might also depend on sensations experienced in the time after eating. Møller (2015) argue that reward from eating depends also on mental and bodily wellbeing experienced after a meal, and that this focus is practically untouched in the scientific exploration of Food Reward [28]. There is a need to extend the concept of affective reward in human reward processes [17], to move further and beyond the actual eating event in seeking to quantify reward, wellbeing, pleasure, food joy and satisfaction derived from eating [28]. This gives rise to incorporating Food Pleasure to our post-ingestive experiences, as well as to explore how Post-Ingestive Food Pleasure associates to other post-ingestive sensations. Food Pleasure can thus also be a measure after intake and in the time following intake. Food Pleasure as a longer-lasting measure than just at the actual eating event can provide deeper insights into what drives Food Pleasure post intake.

In the present paper, we define Post-Ingestive Food Pleasure (PIFP) as a “subjective conscious sensation of pleasure and joy experienced after eating”, measured directly by asking: “Please rate your sense of joy when thinking of the meal you ate today”. Therefore, PIFP in this study represents explicit enjoyment experienced by the individual consumer. Studying post-ingestive drivers of Food Pleasure, whether it be appetite-related sensations or more bodily or mental sensations, requires measurements of our interoceptive states [28,29]. Interoception defines as the subjective experience of internal signals related to, e.g., satiety, hunger, heat, pain, energy, visceral and muscular sensations [29,30,31,32]. Interoceptive states also function as a basis for self-awareness and subjective feelings, representing a conscious evaluation of “how I feel” [28,29]. Including different interoceptive states appeals to consumers’ ability to introspect at a conscious level and contributes to an in-depth picture of the extended appetite experience [28,33].

The overall purpose of this research study was to study post-ingestive sensations as drivers of PIFP in a cross-cultural comparative study between Denmark and China. Investigating the post-ingestive consumer experience including PIFP provides valuable novel insights into the more extended eating experience going beyond the momentary eating event. It enables us to study the dynamics and the interrelationships between sensations in the time after eating and provides a clearer picture as to which sensations that are important for PIFP for Chinese and Danish consumers. PIFP and other post-ingestive sensations can act as desired outcome goals for consumers, and these desired goals could guide the individual to regulate one’s eating behaviors in order to obtain the desired goal. This, indeed, serves important knowledge for both academia, food industry and food policy. Product developers, marketing professionals and health advisers can benefit from this knowledge in order to facilitate and promote healthier eating behaviors and thus help people to better navigate in a challenging food environment. Therefore, investigating PIFP offers new ways to understand and predict food choice and intake behaviors. The research specifically aims to:

(1) Study post-ingestive drivers of self-reported Post-Ingestive Food Pleasure (PIFP), and study the development of drivers of PIFP over three hours post intake;
(2) Compare Danish and Chinese consumers in their post-ingestive drivers of self-reported PIFP.

The research included conducting of two in-country consumer studies in Denmark and in China, respectively. We investigated similarities and differences between Danish and Chinese consumers, and it was hypothesized that differences exist between the two cultures with respect to variation in drivers of PIFP. The constructs of PIFP were therefore expected to vary with consumers’ cultural background. Note, data from the Danish in-country study have been used and published elsewhere, but with different aims and analyses [34]. In the present paper, we include Chinese in-country data, partake different aims and analyses and focus on a cross-cultural perspective comparing dimensions of PIFP between Denmark and China.

## 2. Materials and Methods 

### 2.1. Participants and Recruitment

In China, participants (*n*_total_ = 53) were recruited from the University of Chinese Academy of Sciences (UCAS), north of Beijing. In Denmark, participants (*n*_total_ = 48) were recruited from Ollerup Sports Academy, south of Odense. In both studies, participants voluntarily signed up for the research study after advertisement via internal written communication channels. Inclusion criteria in both countries were: being between 18 and 25 years old, being a liker of yoghurt for breakfast, not suffer from any food allergies and being Chinese or Danish in nationality, respectively. Table 1 displays the characteristics of participants from Denmark and China. The study was approved by the Ethics Committee of Institute of Psychology, Chinese Academy of Sciences with the approval number: H18011. In Denmark, ethical approval is not required for this type of study according to the National Committee on Health Research Ethics in Denmark (Section 14 (2) in the Committee Act) [35]. Prior to participation, all participants gave their written consent. 

### 2.2. Procedure and Study Design

Two comparative central-location consumer studies were carried out in Denmark and in China, both with a randomized controlled crossover design. Data collection procedures were executed in identical manners. Data were collected within a reasonable timeframe, in this case within seven months, avoiding any disproportionate period of time between data collection which can hinder comparability [36]. Data were collected in March 2018 in Denmark and in October 2018 in China, with similar weather conditions, avoiding any bias related to seasonal heat or cold weathers during summer or winter. Participants came in for two breakfast sessions on two separate days. Breakfast meals were served in random order across participants. The study began at 7:30 a.m. in the morning, with participants coming in fasting state since 22:00 the night before, and ran for 3 hours until 10:30 a.m. To make sure, all participants consumed the same amounts, the breakfast meals were mandatory intake. Response variables were collected pre intake, immediately after intake and for three hours in 30 minute intervals post intake. Post-Ingestive Food Pleasure and Satisfaction were evaluated post intake, since they refer to sensations after eating. 

### 2.3. Questionnaire

The questionnaire focused on post-ingestive sensations and PIFP, and the specific chosen response variables were developed from existing scientific literature linked to consumer food perception and investigated post-ingestive sensation variables [6,32,37,38,39]. PIFP in the present study was evaluated with focus and emphasis on food joy post intake using the question phrasing: “Please rate your sense of joy when thinking of the meal you ate today”. Response variables were evaluated in randomized order using a visual analogue scale (VAS) via CompuSense^®^ Cloud software (CompuSense Inc., Guelph, Ontario, Canada). Data were thus collected on a continuous scale ranging from 0, anchored “not at all” to 10, anchored “extremely”. In addition, participants answered demographic question including gender, age, weight and height.

Emphasis was made on ensuring linguistic equivalence between the two languages, as well as developing the questionnaire in participants’ native languages. It has been shown that non-native language questionnaires can lead to a preference for neutral answers due to lack of understanding of the question or lack of comfortability with the task in general, whereas native language questionnaires support the respondent to use the full range and to find the concepts more refined [40]. The questionnaires in each country included identical response variables in linguistic equivalent phrasings. For specific phrasings used in the Danish and Chinese consumer studies, see Table 2. To ensure and validate proper translations for cultural equivalence, question phrasings were translated using back-translation [41]. Chinese native speakers translated the questionnaire from English to Chinese, followed by a back-translation to English by another native speaking Chinese researcher. All translators were researchers within food science to ensure that the meaning of the questions were correctly communicated after translations into another language. 

### 2.4. Breakfast Meals

**Figure 1:**
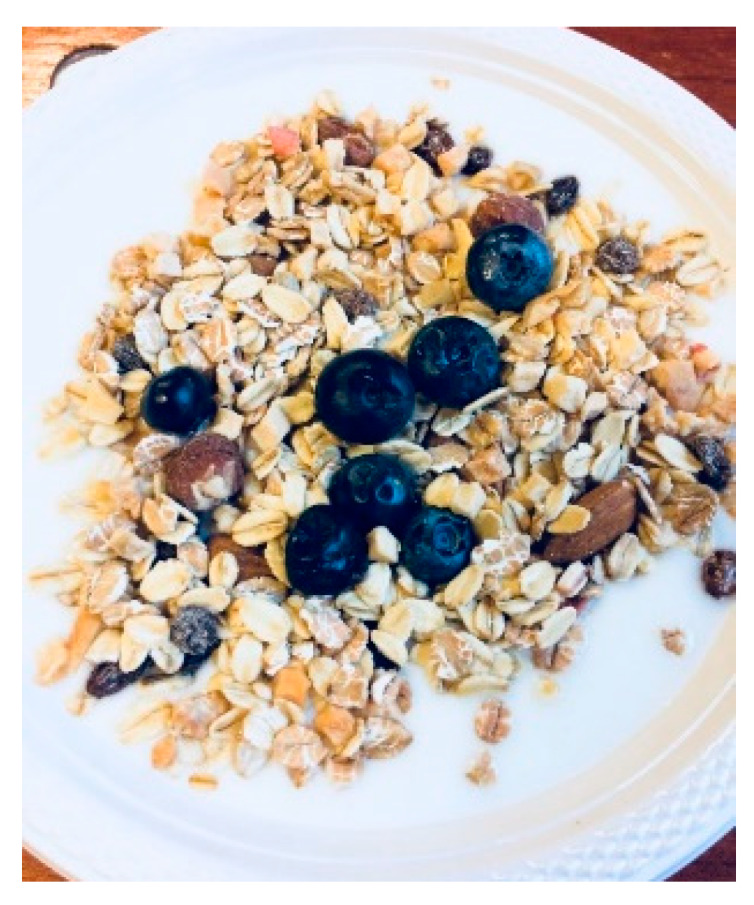
Picture of the breakfast meal (non-flavored yoghurt, muesli, almonds, raisins, fresh blueberries).

The breakfast meals consisted of a non-flavored yoghurt added toppings of plain muesli, natural almonds, raisins and fresh blueberries. The breakfast meals were developed to resemble each other in each country. All ingredients were identical except for the yoghurt and fresh blueberries, which were purchased commercially in China and in Denmark, respectively. Thus, yoghurt and blueberries were locally bought in each country aiding familiarity and fresh produce. In both countries, we selected a non-flavored yoghurt with no added sugar, which was similar in content and calories for both countries. The yoghurt from China contained six more calories per 100g than the Danish yoghurt. To facilitate a span in evaluated response variables, we added whey protein isolate and glucose syrup to the breakfast meals stirred into the yoghurt. The added whey protein isolate (35g/131.5Cal) and added glucose syrup (42.35g/131.5Cal) were the exact same amounts in both countries. For meal content and specific ingredient details, see Table 3. Note, for this paper, the meals served as a tool to span post-ingestive sensations and PIFP, and to facilitate a comparison between Chinese and Danish consumers in post-ingestive drivers of PIFP. Results accounting product/meal differences have been published elsewhere [34]. The test meals were made following standardized procedures to ensure validity and standardization. The visual look of the breakfast meals were identical in China and in Denmark, and can be seen from the picture in Figure 1. 

### 2.5. Data Analysis

Subjective reports on weight and height were used to calculate BMI: weight (kg)/height (m)^2^. Repeated Measures Analysis of Variance (ANOVA) was performed separately for each country to analyze dynamics in post-ingestive sensations over time. *p*-values ≤ 0.05 were considered statistically significant, and Tukey’s Honest Significant Differences (HSD) test was applied for pairwise comparisons between time points. Effect sizes were examined using Cohen’s *d* values [42]. Partial Least Squares Regression (PLSR) models were applied to study drivers of PIFP in Denmark and in China, separately. This approach has also been employed by other researchers for Sensory and Consumer Science data [39,43]. PLSR deals with multi-collinearity between explanatory variables as well as takes into account the latent co-variance structure between PIFP (Y-variable) and explanatory post intake variables (X-variables). Data were centered and reduced, and all models were full cross-validated using Jackknife leave-one-out (LOO) validation. A cumulative Q^2^ index above 0.5 was considered a good predictive quality of the models. Variable Importance in Projection (VIP-score) was analyzed to determine the influential variables on PIFP. Only VIP-scores above 0.8 were considered influential variables as defined by, e.g., [44,45,46]. As described by [47], a strategy to select subsets of variables is discarding of variables with small VIP values [45,47]. Therefore, and for visualization, the plots depicted in the manuscript only displays variables that the models found influential on PIFP, discarding small VIP values. PLSR models were applied across products (across breakfast A and B, thus not accounting meal differences) and separately for China and Denmark. PLSR models and ANOVA models were applied at the time points ‘immediately post intake’ as well as ‘three hours post intake’, to reflect the longest interval and variation in data and to represent the post-ingestive effects for the whole study period. All PLSR models were carried out using XLSTAT by Addinsoft, version 2019.2. (XLSTAT, Long Island, NY, USA) [48]. 

## 3. Results

### 3.1. Dynamics in Post-Ingestive Sensations

Significant main effects of time between the time points ‘immediately post intake’ and ‘three hours post intake’ was seen for 15 post-ingestive variables in Denmark and for 10 post-ingestive variables in China, see Table 4. In general, for both countries, Hunger, Desire-to-eat, Sweet desire, Salty desire, Fatty desire and In need of food significantly increased over three hours, whereas Satiety significantly decreased. Only in China, Overall, Mental and Physical wellbeing significantly increased over three hours, whereas the same variables significantly decreased in Denmark over three hours. In China, no time effects were seen for Energized, Relaxation, Concentration, Sleepiness, Satisfaction and PIFP, whereas only Sleepiness in Denmark did not show a significant time effect. In both countries, Hunger, Satiety, Desire to eat and In need of food yielded the largest effect sizes for time differences. Table 4 shows the least squares (LS) means across products at the two time points for each country. Level of significance, F values and Cohen’s d values for effect sizes are included for each country separately.

### 3.2. Post-Ingestive Sensations Driving Post-Ingestive Food Pleasure in China and in Denmark

Partial Least Squares Regression models were employed to determine the main drivers of PIFP amongst the included fifteen post-ingestive sensations in each country at two different time points; immediately post intake and three hours post intake. All models showed good predictive quality, defined by how well the model fits the observed values (goodness of fit). Accordingly, from the Chinese data, the model quality presented a cumulative Q^2^ index of 0.7 and 0.6; at time immediately post intake and three hours post intake, respectively. From the Danish data, the model quality presented a cumulative Q^2^ of 0.5 and 0.9 immediately post intake and three hours post intake, respectively. Analysis of Variables Importance in Projection (VIP-scores) revealed specific post-ingestive sensations to be drivers of PIFP. In China, the following post-ingestive sensations were main drivers of PIFP: Satisfaction, Mental wellbeing, Overall wellbeing, Physical wellbeing, Energized, Concentration and Relaxation. These post-ingestive sensations remained influential drivers from immediately post intake and until three hours post intake. In Denmark, immediately post intake, the following post-ingestive sensations were main drivers of PIFP: Satisfaction, Mental wellbeing, Overall wellbeing, Physical wellbeing, In need of food, Desire to eat, Satiety and Hunger. Three hours post-intake, the analysis revealed two additional post-ingestive sensations to become influential drivers (VIP-scores >0.8), namely Energized and Concentration. Table 5 shows the VIP-scores from the PLSR models for each country at the two time points. 

**Figure 2:**
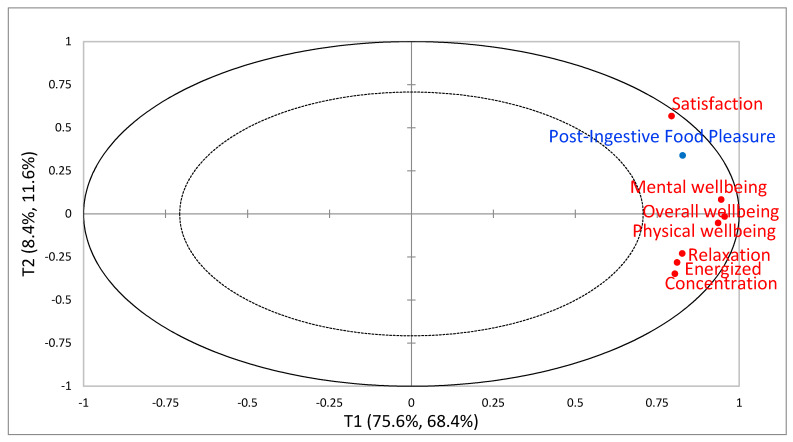
Partial Least Squares Regression (PLSR) correlation loadings plot from the Chinese consumer study immediately post intake, with Post-Ingestive Food Pleasure as dependent variable (Y) and Post-ingestive sensations as explanatory variables (X). Only explanatory variables with VIP-scores >0.8 are included. The plot displays component 1 (X explained variance: 75.6%, Y: 68.4%) vs component 2 (X explained variance: 8.4%, Y: 11.6%).

**Figure 3:**
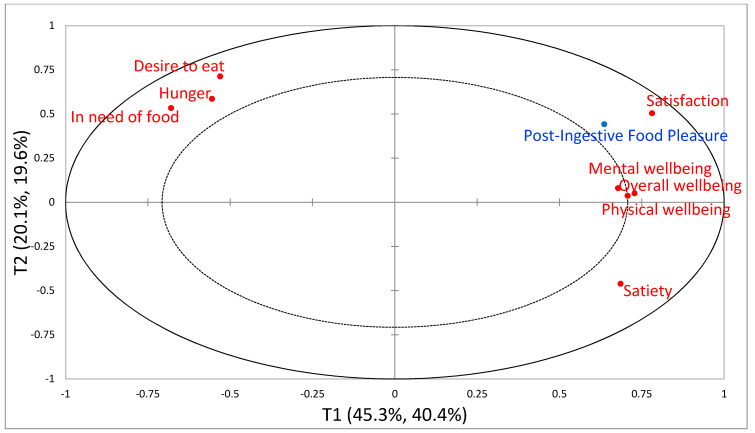
Partial Least Squares Regression (PLSR) correlation loadings plot from the Danish consumer study immediately post intake, with Post-Ingestive Food Pleasure as dependent variable (Y) and Post-ingestive sensations as explanatory variables (X). Only explanatory variables with VIP-scores >0.8 are included. The plot displays component 1 (X explained variance: 45.3%, Y: 40.4%) vs component 2 (X explained variance: 20.1%, Y: 19.6%).

The relationships between variables ‘immediately post intake’ are visually illustrated in Figure 2 and Figure 3 for China and Denmark, respectively. The relationships between variables at ‘three hours post intake’ show the same overall relations (plots not shown). For easing visualization, only influential variables (VIP-scores >0.8) are included in the figures. From the Chinese results (Figure 2), the PLSR model explained in total 80% for the dependent variable (Y) and 84% for the explanatory variables (X). The first component explained 68.4% and 75.6% of Y- and X-data, respectively, and the second component explained 11.6% and 8.4% of Y- and X-data, respectively. Interpreting the plot in Figure 2, we see that component 1 explained the majority of variance in data displaying all the influential post-ingestive sensations (VIP-scores > 0.8) positively correlated with PIFP. Component 2 was somewhat explained by Satisfaction and PIFP, but with low explained variance compared to component 1. From the Danish results (Figure 3), the PLSR model explained in total 60% for the dependent variable (Y) and 65.4% for the explanatory variables (X). The first component explained 40.4% and 45.3% of Y- and X-data, respectively, and the second component explained 19.6% and 20.1% of Y- and X-data, respectively. Overall wellbeing, Mental wellbeing and Physical wellbeing contributed the most in explaining the first component, whereas Desire to eat, Hunger, In need of food and Satiety contributed the most in explaining component 2 dividing the appetite sensations into two, with Satiety opposite the other three appetite sensations and negatively correlated with them. Satisfaction and PIFP were shown to contribute to explaining both components, however, mainly the first component. 

## 4. Discussion

### 4.1. Cross-Cultural Differences in Drivers of Post-Ingestive Food Pleasure

This comparative research aimed to study and compare post-ingestive sensations as drivers of PIFP in Denmark and in China. Key results revealed both cross-cultural similarities as well as cross-cultural difference in post-ingestive drivers of PIFP in Denmark and in China. For both Danish and Chinese consumers, the post-ingestive variables Satisfaction, Mental wellbeing, Overall wellbeing and Physical wellbeing were highly influential on PIFP with VIP-scores > 1. This suggests a somewhat similar structure in the cultural constructs of Food Pleasure post intake and to some degree, a common conceptualization of PIFP in both countries (further elaborated in Section 4.2.). However, results also showed that other and different dimensions of post-ingestive sensations explained consumers’ subjective PIFP in each country. Danish consumers perceived appetite-related sensations such as Satiety, Hunger, Desire-to-eat and In-need-of-food to be influential on PIFP, and this was not the case for Chinese consumers. On the contrary, in China, the more vitality- and energy-related post-ingestive sensations such as Relaxation, Energized and Concentration were found to be drivers of PIFP. 

Importantly, these results thus indicate that Chinese and Danish consumers seem to experience and associate PIFP via different dimensions. Other studies, conducted in European countries, report appetite sensations to influence Satisfaction. For instance, Boelsma et al. (2010) reported that Satiety relate particularly to feelings of Satisfaction [49]. Andersen et al. (2017, 2015) found that main positive drivers of Food Satisfaction included Hunger, Fullness and Energy for Danish consumers [39,43]. Researching Food Reward, Rogers and Hardman (2015) demonstrated Hunger to independently have an effect, this with UK consumers [19]. They, however, measured Food Reward as Desire to eat a portion, defined as the momentary value of a food at the time of ingestion, and not Food Pleasure as a post intake measure such as in this study. The present results from the Danish study agree with above mentioned studies, that appetite sensations such as Hunger and Satiety demonstrate influential drivers in our perception of food. However, the mentioned research studies focused mainly on Food Satisfaction and not PIFP as evaluated in this study.

The question arises whether Danish people are more driven by the homeostatic aspects of appetite such as Hunger and Satiety than the Chinese people are. Furthermore, one might interpret that Chinese people have a different perspective in relation to PIFP with more focus on vitality terms, including Energy and Relaxation. It seems that the Danish consumers experience PIFP as more related to the body’s physical needs, whereas the Chinese consumers experience PIFP as more connected to the body’s ‘mental’ needs. Previous research has also reported differences in the values associated to food and eating between Western countries and China [5,6], e.g., results have pointed towards cultural differences in the way we associate for instance wellbeing and ‘feeling good’. Sulmont-Rossé et al. (2019) report cross-cultural perspectives on ‘feeling good’ in the context of food using qualitative approaches. Their results demonstrated that ‘feeling good’ was associated to emotional, physical (health-related) and social dimensions across countries. However, when specifically looking at China, they found that Chinese people expressed fewer words related to specific food items than other countries in a word association task, but instead provided more mentions related to happiness, joy and enthusiasm. Oppositely, mentions associated to nutrition and healthy diet tended to be more frequent in Western countries [5]. Furthermore, exploring cross-cultural associations with food-related wellbeing, Ares et al. (2016) found the biggest country difference for emotional and spiritual aspects of wellbeing, with especially Chinese people integrating an aspect of nature in association to food and wellbeing [6].

In order to try to understand such cultural differences, it is essential to look at cultural values in broader perspectives. Ma (2015) describes food in China to represent social status and symbolic meaning [50]. In Denmark, food is often more fixated around nutrient content, and whether the food is good or bad for your physical health [51]. Hofstede (1980, 2001) has proposed a model where cultures are compared based on values. The model includes six dimensions: Power distance, Uncertainty Avoidance, Individualism/Collectivism, Masculinity, Long term orientation and Indulgence with index scores ranging from 0-100 [52,53]. Large difference have been reported amongst many countries, and it is evident that, within Hofstede’s framework, cultural differences also exist between Denmark and China. Denmark and China especially differ for the dimensions Individualism (China 20, Denmark 74) and Long term orientation (China 87, Denmark 35)—data retrieved from [54]. China is characterized as a collectivistic culture and Denmark as an individualistic culture. This implies greater focus on harmony and sense of belonging to larger environments in China rather than the individual environment in Denmark. Furthermore, China scores high in the Long term orientation index, referring to a greater importance put on future events rather than the present events [52]. These large value differences between China and Denmark might help us to understand differences in eating behaviors as well. This type of approach can contribute to our understanding of the cultural determinants of differences in food-related behaviors [55]. Nevertheless, care should always be taken in the generalizing of results when comparing and interpreting cross-cultural differences. 

### 4.2. Post-Ingestive Food Pleasure, Satisfaction, and Wellbeing Associations

PIFP demonstrated to have the same four main drivers in both China and in Denmark. Especially Satisfaction revealed to be the biggest driver of PIFP in both countries, regardless of time point, this with VIP-scores as high as 2.6. Moreover, the three wellbeing variables (Overall, Mental and Physical wellbeing), also proved to drive PIFP with big impact in both countries. This indicates that variables such as Satisfaction and subjective Wellbeing can be seen as holistic responses comparable with PIFP (as defined in this study). Supporting this argument, Ares et al. (2015) studied consumer’s association with wellbeing in a food-related context, and found that wellbeing was mainly associated with calmness, happiness and satisfaction [7]. In a qualitative study on consumer reflections on post-ingestive sensations, Duerlund et al. (2019) found pleasure and feeling good to be part of consumers’ perceptions of post-ingestive wellbeing [32]. Furthermore, post-ingestive Psychological Wellbeing was found to be part of Food Satisfaction, in a study from Andersen et al. (2017) [43]. Furthermore, in a study from Sulmont-Rossé et al. (2019), the effects of consuming food included emotional aspects when conceptualizing feeling good. Particularly, consumers refereed to positive emotions such as happy, enthusiastic and satisfied [5]. The present results together with above-mentioned research, supports the link and associations between PIFP, Satisfaction and Wellbeing. 

Interestingly, all these holistic variables; Satisfaction, Overall wellbeing, Mental wellbeing, Physical wellbeing and PIFP, demonstrated to either significantly increase or stay the same over three hours in China, but conversely significantly decreased in Denmark. Hence, we here see a cultural difference in the development of these holistic sensations over time, specifically with a longer-lasting effect in China compared to Denmark. This could indicate a somewhat disconnect between food and the holistic concepts in China compared to Denmark. As demonstrated by Sulmont-Rossé et al. (2019), China associated ‘feeling good’ with emotional and hedonic dimensions rather than to specific food or beverages items, whereas Western countries more often associated it with specific food items [5]. This might help explain the present differences in the development of holistic sensations in China compared to Denmark. 

PIFP was in this study considered and defined as a “subjective conscious sensation of pleasure and joy experienced after eating”, with emphasis on a rewarding sensation of joy lasting after eating rather than reward (liking or desire to eat) whilst eating. PIFP thus included aspects of joy and pleasure lasting after eating with a memory of the food eaten. Memory for recent eating and Food Pleasure are linked. We can for instance recall from memory how enjoyable a food was, which then can also influence the additional after effects from eating [56]. Hence, we cannot be entirely sure how consumers rated PIFP, whether they evaluated joy right NOW relative to the food they ate, or joy with the food WHEN they ate it. Seen in a Denmark-China perspective, and interpreting the fact that PIFP in Denmark decreased over three hours, something could indicate that Danish consumers focus a lot on the sensation they have in their body right now, i.e., that they sense more joy with the food right after it is eaten than after three hours. In Denmark, it seems that before consumers experience PIFP, the food must meet some physical bodily needs. These are often related to the food’s ability to keep one sated, but it must also ensure to keep one energized for daily chores and to maintain one’s concentration. As time passes, the food is ‘put to the test’ by the consumer in order to keep his/her energy level up and to facilitate concentration. This could explain why Energized and Concentration become explanatory for PIFP in Denmark three hours after eating. In China, on the other hand, the ratings of PIFP were constant over time, which indicates that they do not have the same requirements for food to satisfy some physical bodily needs, but that food has a more mental effect longer term. This indicates that the Chinese consumes rated PIFP as the joy with the food when they ate it, and that this joy is not vulnerable to change over time, maybe because it is not tied to the same requirements for food to meet certain needs as in Denmark. 

The fact that different sensations influenced PIFP in China and in Denmark in this study, suggest some cross-cultural differences in the way we perceive food and the association we have with food. Understanding the differences and elucidating ‘why’ is multi-faceted. One thing is for sure, cross-cultural research is becoming more important as a result of rapid globalization, also within Sensory and Consumer Science [3,55,57]. As we know, culture is one of the main factors underlying how we assess and choose food [1], and the present results can illuminate what is important when exposing consumers to new products in new markets. It is clear that more research is warranted to elucidate cross-cultural differences in our eating behavior and perception of food. 

As mentioned by Møller (2015), Food Reward comes in many and different disguises, both in terms of immediate liking and motivational wanting of a food whilst eating, but also as a longer lasting feeling of for instance wellbeing after a meal. Both aspects are included and suggested to be valid components of Reward in Berridge and Robinson’s powerful model of Reward components [12]. In this study, we situated and considered PIFP as part of the Affect category within reward with focus on conscious pleasure derived from eating. Quantifying joy and pleasure obtained from eating food as a Food Pleasure measure provides pertinent and relevant output, and, importantly, the results take us beyond the processes only active during the actual eating event [12,28]. 

### 4.3. Limitations

An important aspect to consider when interpreting the results from this cross-cultural comparative study between China and Denmark is the composition and nature of the participants in each country. Recruitment resulted in unequal gender distribution with particularly more males than females in Denmark. In China, gender was more equally distributed, but with a slight majority of female participants. Quota sampling, rather than convenience sampling, is recommended for comparing consumer perceptions across cultures [55]. Participant characteristics, therefore, also differed, with higher average weight and height in Denmark than in China. However, this, in general, reflects the average size of the people living in the two countries as well. Furthermore, participants in Denmark were recruited from a Sports Academy, possibly placing, even more than normal, emphasis on physical performance, which could be reflected in the results around PIFP. 

Standardization between the served breakfasts meals in the two countries were attained with the same amounts served in Denmark and in China. With this said, some of the Danish participants commented on too small amounts, whereas some of the Chinese participants commented on too large amounts. This could, certainly, also be explained by the before-mentioned differences in weight and height for the participants, with larger males naturally needing greater amounts of food. Different yoghurts were used in each country in order to aid fresh produce and familiarity. However, a perspective to consider here is the familiarity in general for yoghurt as a breakfast meal. Familiarity could have affected PIFP or other ratings in unknown ways in this study. Participants, though, were recruited being likers of yoghurt for breakfast. That being said, commercially available yoghurts in China consists mainly of flavored yoghurts, whereas Danish people perhaps are more used to non-flavored yoghurts. Additionally, our results, whilst representative for young consumers, may differ when considered in relation to the general population in both countries. Furthermore, whilst our results apply to eating breakfast, results may vary if applied in other contexts such as lunch or dinner. Therefore, more confirmatory research studies are needed with differing population groups, as well as application in different contexts, in order to establish the generalizability of the results from the present study. 

In cross-cultural research, it is important to acknowledge possible cultural differences in response style, because culture is known to have a strong influence on how people use scales to answer questions [55,58]. Especially extreme response style, middle response style, and acquiescence response style are common response styles to acknowledge and be aware of [40,55,59,60]. An extreme response style can be characterized by the tendency to use the end-points more frequently than for instance acquiescence response style characterized by the tendency to respond with agreement or affirmation. Especially Likert scales and semantic differential scales (i.e., good to bad, dirty to clean) are vulnerable to response style tendencies. Furthermore, scale anchors indicating the degree of agreement or importance are more vulnerable to acquiescence response style in cultures with high Power distance and Collectivism [40]. In this study, we did not use Likert scales indicating the degree of agreement or importance. We used intensity scales with semantic similar anchor points (i.e., “not at all energetic” to “extremely energetic”). Different approaches are used to investigate response style tendencies, where the most commonly used are frequency or percentage of answers in particular categories of a scale [40,59,60], or a multisample confirmatory factor analysis if scales are to be directly compared [55,61,62]. Calculating percentages of answers in the top 25% and bottom 25% of the intensity scales in both countries, showed equal distribution of answers in both low, middle, and high end of the intensity scale for both countries. No formal multisample confirmatory factor analysis was conducted to test direct scale comparability, since we did not directly compare the scales. All analyses were conducted separately for each country with no direct comparisons in raw data. Actions can be taken to account for difference in response style, e.g., standardization and/or centering of data. However, it should be taken into account that standardization can potentially remove some of the true differences among cultures [40]. It is, nevertheless, important to acknowledge possible hidden differences in response styles for the two countries, and in general consider potential cultural bias in methodology when doing cross-cultural research [36,55]. Unknown differences in response style and understanding of terms might have influenced the results in these studies [60], but could very well also be a true difference in cultures for the post-ingestive experiences.

### 4.4. Research Contribution and Future Perspectives

The present research findings contribute new knowledge about the cultural differences between China and Denmark. Specifically, we contribute to a better understanding of the differences in consumers’ post-ingestive experiences including PIFP and its drivers in each country. This knowledge helps to unravel and recognize some aspects of cultural differences when addressing food culture and eating behaviors. A key contribution to the cross-cultural research field, and a major advantage of this study, is the actual serving of food and evaluation of consumers’ perceptive responses to actual intake. The two studies thus provide centrally collected data in each country with native consumers and in-country behavior. Other cross-cultural research often contributes with knowledge collected online with no serving or eating of food, and/or with expatriates as consumers who no longer reside in their original culture [2]. 

The present results and knowledge can support researchers as well as the food industry to a better understanding of the cultural differences between Denmark and China. Particularly, food companies could use this knowledge when applying and introducing new products to new markets and cultures. Furthermore, continuing conducting cross-cultural research is important and relevant. The contribution to the scientific field and to fundamental research is highly important, since we still have limited knowledge as to how we perceive food and associate food between cultures in today’s rising globalization.

Future perspectives for this particular research focus within PIFP and in the Sensory and Consumer Science area should include alternative ways to measure and collect data in cross-cultural research. Examples could include the application of more implicit measures, such as behavioral, indirect approaches to scaling, and biometric measures, which would provide usable data. Targeting Food Pleasure and other aspects of Food Reward from different angles including both explicit and implicit measures could add to the validation and application of the concept. This would require multidisciplinary approaches combining Sensory and Consumer science with, e.g., neuroscience, biology, psychology and human nutrition utilizing a multimodal approach.

## 5. Conclusions

This cross-cultural research study aimed to compare Danish and Chinese consumers in their post-ingestive drivers of Post-Ingestive Food Pleasure (PIFP). The work involved conducting two in-country consumer studies in Denmark and in China, respectively, measuring self-reported PIFP after eating together with other post-ingestive sensations. Key results revealed that for both Danish and Chinese consumers, the post-ingestive variables Satisfaction, Mental wellbeing, Overall wellbeing and Physical wellbeing were highly influential on PIFP. This suggests a somewhat similar structure in the cultural constructs of PIFP in both countries. However, the results also revealed that disjointed and different dimensions of post-ingestive variables drove consumers’ PIFP in each country. Danish consumers perceived appetite-related sensations such as Satiety, Hunger, Desire-to-eat and In-need-of-food to be influential on PIFP, which was not the case for the Chinese consumers. On the contrary, in China, the more vitality- and energy-related post-ingestive variables such as Relaxation, Energized and Concentration were found to be drivers of PIFP post intake. These results resonate with our research hypothesis showing distinct subtleties in the cultural constructs of PIFP in Denmark and in China. 

These findings serve relevance to various areas. The contribution to science is highly important, since we still have limited knowledge as to how we perceive and associate food between cultures. Moreover, the food industry can indeed benefit from taking into account consumers’ both physical and mental sensations when designing products for different markets, including knowing that cultural differences exist as to which sensations drive Food Pleasure after eating. Investigating the post-ingestive consumer experience thus matters, because it provides valuable novel insights into the elaborated eating experience, which goes beyond the momentary eating event. 

Cross-cultural differences exist in the way we perceive food and the association we have with food, and the constructs of PIFP also vary with consumers’ cultural background. Understanding and explaining ‘why’ these differences exist is multi-faceted and they do not have a single answer. One thing is certain, cross-cultural research becomes extra relevant in the context of rapid globalization, especially within sensory and consumer science. Undoubtedly, more research is needed to elucidate cross-cultural differences in our eating behavior and perception of food. 

## Figures and Tables

**Table 1:** Participant characteristics from the Danish and Chinese central-location studies.

Characteristics	China	Denmark
*n*_total_	53	48
Males/females	24/29	31/17
Age (years)	22 ± 1.1 (20–25) *	20.4 ± 1.1 (18–25) *
Weight (kg)	60.3 ± 10.8 (41–95) *	71.5 ± 12.0 (51–108) *
Height (cm)	168.6 ± 7.2 (154–183) *	175.7 ± 8.5 (162–192) *
BMI ^1^ (kg/m^2^)	21.1 ± 2.7 (17.3–33.6) *	23.0 ± 2.4 (19–30.5) *

* Mean ± standard deviation (range); ^1^ BMI= body mass index

**Table 2:** Response variables with Danish, English, and Chinese phrasings as used in the questionnaires. Data were collected on a continuous visual analogue scale (VAS) ranging from 0 to 10.

Variable	English Phrasing	Chinese Phrasing	Danish Phrasing
Hunger	“How hungry are you right now?”	“您现在有多饿?”	“Hvor sulten er du lige nu?”
Satiety	“How full are you right now?”	“您现在有多饱?”	“Hvor mæt er du lige nu?”
Energized	“How energetic are you right now?”	“您现在精力有多充沛?”	“Hvor energisk er du lige nu?”
Relaxation	“How relaxed are you right now?”	“您现在有多放松?”	“Hvor afslappet er du lige nu?”
Concentration	“How is your concentration right now?”	“您现在精神有多集中?”	“Hvor koncentreret er du lige nu?”
Sleepiness	“How sleepy are you right now?”	“您现在有多累?”	“Hvor træt er du lige nu?”
Satisfaction	“How satisfied are you with the breakfast meal you ate today?”	“您对今天的早餐多满意?”	“Hvor tilfreds er du med det måltid du har spist i dag?”
Overall wellbeing	“Please rate your overall wellbeing as you feel it right now”	“请立即给您现在的总体舒适状况打分”	“I hvor høj grad fornemmer du en generel velvære lige nu?”
Physical wellbeing	“Please rate your physical wellbeing as you feel it right now”	“请您凭第一感觉对自己现在的身体舒适状况打分”	“Hvor fysisk veltilpas er du lige nu?”
Mental wellbeing	“Please rate your mental wellbeing as you feel it right now”	“请您凭第一感觉给自己现在的心情舒畅状况打分”	“Hvor mentalt veltilpas er du lige nu?”
Desire to eat	“How much do you desire to eat something right now?”	“您现在有多想吃点东西呢?”	“Hvor stor er din lyst til noget at spise lige nu?”
Sweet desire	“How much do you desire to eat something sweet right now?”	“您现在有多想吃些甜的食物?”	“I hvor høj grad har du lyst til noget sødt lige nu?”
Salty desire	“How much do you desire to eat something salty right now?”	“您现在有多想吃些咸的食物?”	“I hvor høj grad har du lyst til noget salt lige nu?”
Fatty desire	“How much do you desire to eat something fatty right now?”	“您现在有多想吃些油腻的食物?”	“I hvor høj grad har du lyst til noget fedt lige nu?”
In need of food	“How much do you need food right now?”	“您现在有多需要食物?”	“I hvor høj grad mangler du noget mad lige nu?”
Post-Ingestive Food Pleasure	“Please rate your sense of joy when thinking of the meal you ate today”.	“请给您在回想今日所进食食物的时候的愉悦程度打分”	“I hvor høj grad fornemmer du en glæde ved den mad du har spist i dag?”

**Table 3:** Contents of the breakfast meals.

Food Ingredient	Breakfast A	Breakfast B
	Amount (g)/Calories (Cal)	Amount (g)/Calories (Cal)
Yoghurt in Denmark ^1^	300/123	300/123
Yoghurt in China ^2^	300/141	300/141
Lacprodan ^3^	35/131.5	-
Glucose syrup ^4^	-	42.35/131.5
Muesli ^5^	30/99	30/99
Almonds ^6^	6/30	6/30
Raisins ^7^	2.5/5	2.5/5
Fresh blueberries ^8^	2.5/5	2.5/5
Total Denmark	376/393	383.35/393
Total China	376/411	383.35/411

^1^ Arla® lactose-free yoghurt natural (Arla Foods, Viby, Denmark); ^2^ JinShi Dai 今时代 low-fat, sugar-free yoghurt containing xylitol (Odward Dairy, Beijing, China); ^3^ Lacprodan® SP-9225 Instant (whey protein isolate) (Arla Foods, Viby, Denmark); ^4^ Dansukker® glucose syrup (Dansukker, Copenhagen, Denmark); ^5^ Kornkammeret muesli (Lantmännen Cerealia A/S, Vejle, Denmark); ^6^ Coop almonds natural (Coop Danmark A/S, Albertslund, Denmark); ^7^ Urtekram Sultana raisins (Urtekram International A/S, Mariager, Denmark); ^8^ Bought from a local supermarket the day before test day—origin unknown.

**Table 4:** Least squares means ± standard deviations (China: *n* = 53, Denmark: *n* = 48) across products immediately post intake and three hours post intake for all post-ingestive response variables.

	China					Denmark				
	Immediately Post Intake	Three Hours Post Intake	*p*	F	d ^1^	Immediately Post Intake	Three Hours Post Intake	*p*	F	d ^1^
Hunger	1.81^a^ ± 1.8	4.52^b^ ± 2.4	***	121.8	1.3	4.36^a^ ± 2.6	7.90^b^ ± 1.5	***	205.3	1.7
Satiety	7.61^a^ ± 2.1	4.33^b^ ± 2.4	***	195.2	1.5	5.66^a^ ± 2.0	1.99^b^ ± 1.6	***	267.6	2.0
Energized	6.52 ± 2.0	6.37 ± 2.0	ns	-	-	4.31^a^ ± 1.6	3.72^b^ ± 1.6	**	10.9	0.4
Relaxation	6.70 ± 2.2	7.06 ± 1.7	ns	-	-	5.70^a^ ± 1.7	4.66^b^ ± 1.8	***	20.2	0.6
Concentration	6.96 ± 1.9	6.67 ± 1.8	ns	-	-	4.73^a^ ± 1.4	3.92^b^ ± 1.5	***	17.7	0.6
Sleepiness	3.17 ± 2.0	3.75 ± 2.1	ns	-	-	5.05 ± 1.9	4.86 ± 2.0	ns	-	-
Satisfaction	5.72 ± 2.4	6.05 ± 2.2	ns	-	-	4.48^a^ ± 2.1	3.28^b^ ± 2.0	***	52.5	0.6
Overall wellbeing	6.19^a^ ± 2.2	6.79^b^ ± 1.8	**	9.1	0.3	5.37^a^ ± 1.5	4.39^b^ ± 1.9	***	23.8	0.6
Physical wellbeing	6.00^a^ ± 2.2	6.65^b^ ± 1.9	**	10.5	0.3	5.24^a^ ± 1.5	4.16^b^ ± 1.9	***	29.8	0.6
Mental wellbeing	6.26^a^ ± 2.1	6.93^b^ ± 1.8	***	14.8	0.3	5.36^a^ ± 1.6	4.44^b^ ± 1.7	***	26.0	0.6
Desire to eat	2.38^a^ ± 2.3	4.83^b^ ± 2.5	***	89.3	1.0	4.73^a^ ± 2.5	8.15^b^ ± 1.4	***	202.2	1.7
Sweet desire	2.57^a^ ± 2.3	3.51^b^ ± 2.5	***	23.2	0.4	4.02^a^ ± 2.3	5.12^b^ ± 2.4	***	19.7	0.5
Salty desire	3.14^a^ ± 2.8	3.94^b^ ± 2.5	**	11.9	0.3	2.95^a^ ± 2.0	3.92^b^ ± 2.5	***	19.1	0.4
Fatty desire	2.28^a^ ± 2.4	2.96^b^ ± 2.4	**	10.9	0.3	2.50^a^ ± 1.9	3.74^b^ ± 2.6	***	37.5	0.5
In need of food	2.28^a^ ± 2.1	4.79^b^ ± 2.5	***	100.8	1.1	4.61^a^ ± 2.5	8.01^b^ ± 1.4	***	237.0	1.7
Post-Ingestive Food Pleasure	5.93 ± 2.4	6.16 ± 2.1	ns	-	-	4.19^a^ ± 2.2	3.08^b^ ± 1.9	***	35.0	0.5

Means with different superscript (^a,b^) within each country differ significantly (Tukey *p* < 0.05); *** *p* < 0.0001, ** *p* <0.01, ns = no significant time difference; ^1^ Effect size (Cohen’s d); data were collected on a continuous visual analogue scale (VAS) ranging from 0 to 10.

**Table 5:** Variable Importance in Projection (VIP-scores) of Post-Ingestive Food Pleasure for all fifteen post-ingestive sensations immediately post intake and three hours post intake. Variables are computed in descending order according to VIP-scores immediately post intake.

China			Denmark		
Post-Ingestive Sensation	Immediately Post Intake	Three Hours Post Intake	Post-Ingestive Sensation	Immediately Post Intake	Three Hours Post Intake
Satisfaction	**1.7**	**2.4**	Satisfaction	**2.6**	**2.6**
Mental wellbeing	**1.6**	**1.4**	Mental wellbeing	**1.3**	**1.0**
Overall wellbeing	**1.5**	**1.1**	Overall wellbeing	**1.3**	**1.1**
Physical wellbeing	**1.5**	**1.1**	Physical wellbeing	**1.0**	**1.0**
Energized	**1.2**	**1.3**	In need of food	**1.0**	**0.9**
Concentration	**1.2**	**1.1**	Desire to eat	**0.9**	**1.0**
Relaxation	**1.1**	**1.3**	Satiety	**0.9**	**0.9**
Sleepiness	0.6	0.7	Hunger	**0.8**	**1.1**
In need of food	0.6	0.5	Relaxation	0.6	0.6
Satiety	0.6	0.6	Energized	0.6	**1.2**
Desire to eat	0.5	0.6	Salty desire	0.6	0.2
Salty desire	0.6	0.6	Sleepiness	0.5	0.1
Hunger	0.5	0.5	Sweet desire	0.5	0.2
Sweet desire	0.5	0.5	Concentration	0.5	**1.1**
Fatty desire	0.3	0.6	Fatty desire	0.5	0.2

VIP-scores are calculated based on Partial Least Squares Regression (PLSR) models with Post-Ingestive Food Pleasure as dependent variable (Y) and post-ingestive variables as explanatory variables (X). A VIP-score > 0.8 is considered significantly influential on Post-Ingestive Food Pleasure and highlighted in **bold** in this table.
